# No Change in Inhibitory Control or P3 Following Different High-Intensity Interval Exercise Modalities

**DOI:** 10.3390/brainsci12020185

**Published:** 2022-01-29

**Authors:** Eric S. Drollette, Megan N. Johnson, Caroline C. Meadows

**Affiliations:** 1 Department of Kinesiology, University of North Carolina at Greensboro, Greensboro, NC 27402, USA; mnjohnson@uncg.edu; 2 Department of Kinesiology, School of Sciences and Mathematics, Greensboro College, Greensboro, NC 27401, USA; ccmeadow@uncg.edu

**Keywords:** physical activity, cognition, executive function

## Abstract

Acute aerobic high-intensity interval exercise (HIIE) has demonstrated positive effects on inhibitory control and P3 event-related potential (ERP) in young adults. However, the evidence is not well established regarding the effects of different HIIE modalities that incorporate aerobic-resistance training on these cognitive and neurocognitive outcomes. The purpose of this investigation was to examine the transient effects of HIIE-aerobic and HIIE-aerobic/resistance on P3 and Flanker task performance. Participants (*n* = 24; 18–25 years old) completed the Flanker task at two time points (30 min and 85 min) following 9 min of HIIE-aerobic (intermittent bouts of walking and running at 90% of maximal heart rate), HIIE-aerobic/resistance (intermittent bouts of walking and high-intensity calisthenics), and seated rest on three separate counterbalanced days. Results revealed no changes in Flanker performance (i.e., reaction time and response accuracy) or P3 (latency and mean amplitude) following either HIIE conditions compared to seated rest. Together, these data suggest inhibitory control and neuroelectric underpinnings are not affected by different modalities of HIIE at 30 min and 85 min post-exercise. Such findings reveal that engaging in short bouts of different HIIE modalities for overall health neither improves nor diminishes inhibitory control and brain function for an extended period throughout the day.

## 1. Introduction

Researchers have sought to establish a dose–response relation between parameters of acute exercise (i.e., intensity, mode and time) and cognitive outcomes, with the intent of providing a universal ‘prescription’ to achieve optimal cognitive benefits. Indeed, early meta-analytical data suggest that intensity may be a significant moderator of cognitive benefits revealing an inverted-U relation, with the greatest benefits observed following moderate-intensity compared to both low-intensity and high-intensity bouts [1,2,3]. However, additional meta-analytical evidence complicates this assumption, revealing comparable improvements in cognition at high intensity [4] and low intensity [5]. Thus, intensity may not be a sufficient or exclusive moderating factor to account for cognitive improvements following acute exercise. These comparable improvements at both high-intensity and low-intensity suggest that additional factors may also be influential at different intensities. Interestingly, recent research reveals greater cognitive benefits for different modalities (or types) of exercise including high-intensity interval exercise (HIIE) compared to continuous aerobic exercise, low-intensity active stretching and seated rest [6,7,8,9,10,11], suggesting that exercise modality may also be a contributing factor to cognitive benefits. HIIE is an emerging training modality typically characterized by intermixed repeated high-intensity exercise bouts (above the anaerobic threshold) and low-intensity recovery periods that last for a short period [12,13]. Research suggests that short bouts of HIIE provide physiological health benefits comparable to bouts of continuous aerobic exercise of half the duration [14,15,16]. The current study sought to evaluate the effects of different HIIE modalities on temporal dynamics of executive function, specifically inhibitory control and associated neuroelectrical brain-function outcomes in young adults.

Executive function is responsible for selection, scheduling and maintenance of goal-directed behavior [17,18,19]. Inhibitory control is part of executive function and represents the ability to focus on relevant demands and regulate responses to irrelevant distractions [20]. Neuroelectrical measures, including event-related potentials (ERPs), offer additional insight into executive function processes. Specifically, the P3 component is associated with cognitive outcomes of stimulus probability and task relevance [21]. To date, most research evaluating inhibitory control and P3 measures following an acute bout of exercise has focused on continuous aerobic exercise, with data revealing significant behavioral and neuroelectrical improvements immediately following such exercise [22,23,24,25,26,27]. Similar P3 changes have been observed in research exploring alternative exercise modalities, including HIIE. For example, Kao and colleagues [9] had participants complete three separate conditions: 16 min of HIIE (1 min running at 90% of HR_max_ with intermittent 1 min bouts of walking); continuous aerobic running (treadmill walking/running at 70% of HR_max_); and seated rest, undertaken on three separate days and followed by completion of a Flanker task and P3 assessment. Results revealed improvements in performance for HIIE and continuous aerobic exercise compared to seated rest, with greater improvements observed specifically for HIIE. Additionally, a larger P3 amplitude was observed following continuous aerobic running compared to HIIE and rest. However, HIIE demonstrated a shorter P3 latency suggesting improved processing speed and stimulus classification. These data suggest that HIIE is comparable to traditional moderate-intensity exercise when it comes to cognitive benefits, and that modality may be a significant factor that impacts neuroelectrical and behavioral changes following acute exercise.

To date, acute HIIE laboratory protocols have focused primarily on sustained aerobic intervals (i.e., cycling and running). A limitation of this style of research is the modality disconnect compared to typical HIIE protocols performed in real-world settings. That is, a majority of HIIE protocols developed by fitness professionals integrate forms of resistance exercise and calisthenics that are designed as a holistic high-intensity strength and conditioning program. Furthermore, recent research has sought to evaluate the feasibility of effective HIIE protocols that can be accomplished in workplace and university environments, revealing that a combination of aerobic and muscular fitness protocols is preferred and enjoyable for working employees [28] and university students [29]. As such, a more translational approach would be to evaluate modalities of HIIE that match real-world application by incorporating holistic exercise routines with resistance and aerobic components (i.e., HIIE-aerobic/resistance) and to determine whether the previously identified cognitive and neuroelectrical benefits remain. Although the effects of HIIE-aerobic/resistance on inhibitory control and ERP measures have not been explored previously, research evaluating traditional resistance exercise has drawn mixed findings. For example, Brush and colleagues [30] had participants complete low-, moderate- and high-intensity resistance exercise and rest, followed by a battery of executive function measures. Their results revealed improved executive function performance at 15 min post-exercise for high-intensity resistance exercise and at 180 min post-exercise for low and moderate intensities. Conversely, Pontifex and colleagues [31] had young adults complete separate modalities of acute resistance and aerobic exercise (30 min duration) and only observed improvements in executive function immediately following and 30 min after aerobic exercise, with no improvements observed at either time point following resistance exercise. Lastly, Tsai and colleagues [32] compared changes in executive function following high- and moderate-intensity resistance exercise (40 min duration), revealing no significant differences between the two conditions when assessed approximately 5 min following the cessation of the exercise bouts. Together, these findings suggest that resistance exercise modality may not be as efficacious as aerobic activity for cognitive improvements. However, given the positive results of HIIE-aerobic, incorporating elements of resistance movements within HIIE may offer a unique modality that mitigates the lack of conclusive findings in this previous research.

The timing of cognitive assessments following HIIE may also serve as a significant moderator of cognitive changes. That is, research suggests that cognitive performance declines immediately after exercise but improves after a delay period [1]. For example, Tsukamoto et al. [10] reported delayed cognitive benefits for HIIE at 30 min post-exercise, with maintained performance for HIIE, but not for moderate-intensity continuous activity. These data suggest that HIIE may have a delayed influence on cognitive changes compared to aerobic exercise.

The first aim of the present study was to examine the effects of a single bout of HIIE-aerobic and HIIE-aerobic/resistance on inhibitory control performance and neuroelectrical measures of the P3 in young adults. Based on the findings outlined previously, it was predicted that HIIE-aerobic would improve behavioral performance compared to HIIE-aerobic/resistance and seated rest. Regarding the P3, it was predicted that measures would replicate Kao and colleagues [8], with decreased amplitude and shorter P3 latency for both HIIE-aerobic and HIIE-aerobic/resistance compared to rest. The second aim was to explore temporal changes in cognitive performance at extended time points following the cessation of HIIE and seated rest. It was hypothesized that an increase in task performance would be observed following a delay (30 min post-exercise) and that task performance would return to a level comparable to rest for both HIIE-aerobic and HIIE-aerobic/resistance following an extended interval (85 min post-exercise). Lastly, it was predicted that P3 measures would follow a similar trend, with maintained improvements for the HIIE-aerobic, while the amplitude and latency measures for HIIE-aerobic/resistance would return to levels comparable to seated rest after a delay. Such findings may have implications for promoting healthy physical activity behaviors, under time constraints, without interfering with cognitive functioning for an extended period throughout the day.

## 2. Materials and Methods

### 2.1. Participants

Young adults were recruited from the University of North Carolina at Greensboro. All participants provided written consent approved by the Institutional Review Board. Participants were sent the following electronic questionnaires via Qualtrics: a questionnaire concerning their health history and demographics; a Physical Activity Readiness Questionnaire (PAR-Q) [33]; and the Edinburgh Handedness Inventory [34]. Participants were asked to complete these prior to the first day of testing, to ensure right-hand dominance, normal or corrected-to-normal vision based on the minimal 20/20 standard and the absence of any pre-existing physical or neurological health conditions. Thirty young adults between the ages of 18 and 30 years qualified for the study and completed the informed consent. Final analyses were performed using data from twenty-four participants. Their demographic data were as follows: 18 females; average age 21.4 years (18–25 range); 25% black or African American, 54.2% white or Caucasian, 4.2% Alaska Native or American Indian, 8.3% Asian and 8.3% mixed or other. Reasons for missing data included drop-outs after first (*n* = 2) or second (*n* = 3) laboratory visit, poor performance on the Flanker task (<50% accuracy) and insufficient ERP trials in one or more conditions (*n* = 1).

### 2.2. Flanker Task

A modified version of the Erikson Flanker task [35] was utilized in the present study to assess aspects of inhibitory control. Participants responded, using a 4-button response pad (Current Designs Inc., Philadelphia, PA, USA) to match the directionality of a central arrow presented amidst lateral flanking arrows on a computer screen. Stimuli were white arrows presented on a black screen for 100 ms with variable inter-stimulus intervals of 1000 ms, 1200 ms or 1400 ms. Participants were instructed to respond as quickly and accurately as possible to the direction of the center arrow, amidst congruent (> > > > > or < < < < <) and incongruent (> > < > > or < < > < <) trial types. Participants completed three blocks of 108 trials with equiprobable congruency and directionality at 30 min and 85 min following HIIE-aerobic, HIIE-aerobic/resistance and seated rest. Practice included 53 trials at the start of each testing day. Interference scores were calculated as incongruent–congruent for reaction time (RT) and congruent–incongruent for response accuracy.

### 2.3. ERP Recording

EEG activity was recorded from 64 Ag/AgCl sintered electrode sites organized in accordance with the international 10–10 system [36] using a Neuroscan Quick-Cap (Compumedics Neuroscan, Charlotte, NC, USA). Prior to recordings, electrodes were filled with conductive gel and impedance was maintained at <10 kΩ. Online data were referenced to a midline electrode between Cz and CPz with Fz as the ground electrode. To monitor electrooculographic (EOG), vertical (VEOG) and horizontal eye movement (HEOG), supplemental electrodes were placed above and below the left orbit and outer canthus of each eye. Using a Neuroscan SynAmps2 amplifier, online continuous data were digitized at a sampling rate of 1000 Hz, amplified 500 times with a DC to 70 Hz filter, and a 60 Hz notch filter was applied.

Offline data were processed using MATLAB (R2017a) with EEGLAB [37] and ERPLAB [38] toolbox plugins. Data were re-referenced to averaged mastoids (M1, M2). EEG signal was filtered with a high-pass filter (0.1 Hz). Eyeblink artifact was removed utilizing an automated independent component analyses (ICA) procedure. ICA decompositions were performed using the extended infomax algorithm followed by a time series correlation method that compared point-by-point raw VEOG data with separate ICA activation waveforms (i.e., EEG.icaact matrix generated by the ICA procedure). After validating their consistency and temporal match with raw VEOG artifacts in continuous EEG data, no more than two ICA components with a correlation coefficient greater than 0.30 were removed. Data were back-projected without rejected ICA components for further ERP decomposition.

Stimulus-locked epochs were created at −200 ms to 1000 ms encompassing congruent and incongruent trial types. Epochs were baseline corrected using pre-stimulus intervals and low pass filtered at 30 Hz. Epochs were rejected if a moving window peak-to-peak amplitude exceeded 100 mV (100 ms window width and 50 ms window step); if the overall variance of the epoch exceeded ±3 SDs of the mean of local (by electrode site) and global (all electrode sites) accepted epochs; and/or by visual inspection. ERP-averaged waveforms were created separately for trial type (congruent and incongruent), for each condition (HIIE-aerobic, HIIE-aerobic/resistance and seated rest) and at each time point (35 min and 85 min). After artifact rejection, individual ERP waveforms for congruent and incongruent trials across each condition and time were created from an average of 125 epochs (95% CI: 118 to 132). P3 latency was quantified using peak latency, and P3 amplitude was quantified using mean amplitude within the post-stimulus latency window 300 ms to 600 ms.

### 2.4. Procedure

Using a within-participants cross-over design, participants attended the lab on four separate days (with approximately one week between each testing session). Participants were instructed to avoid vigorous physical activity and to maintain typical daily behaviors (i.e., sleep, food and beverage consumption and work/school activities) 12 h prior to testing. During the first visit, participants reviewed the informed consent with the experimenter, completed practice trials for the cognitive task, and performed a cardiorespiratory fitness assessment on a motor-driven treadmill following a modified Balke Protocol [39] to determine maximal heart rate (HR_max_) for the exercise conditions. Days 2, 3 and 4 were counterbalanced across sessions. At the beginning of each session participants were fitted with a heart rate (HR) monitor and completed practice trials for the Flanker task. They then undertook 9 min of the intervention, were fitted with an electroencephalography (EEG) cap, and completed the Flanker task at 30 and 85 min following each intervention condition, while recording continuous EEG measures. These measurement periods were determined based on prior research revealing a delayed cognitive benefit following HIIE [10] with the second period selected to potentially observe a return to baseline in behavioral performance and P3 measures. The HIIE-aerobic protocol, performed on the treadmill, consisted of a 1 min warm up, 3 sets of 1.5 min running at 90% of HR_max_ (determined by cardiorespiratory fitness assessment) separated by 1 min walking, followed by a 1.5 min cool-down walk. Speed and incline were manipulated by the researcher to obtain 90% of HR_max_. The purpose of selecting the timing and duration of each set was to replicate the methodology of prior research revealing changes in inhibitory control and P3 following 9 min of HIIE [8]. The HIIE-aerobic/resistance protocol consisted of a 1 min warm up on the treadmill, 3 sets of 1.5 min of as many rounds as possible (AMRAP: 10 m run, 20 jumping jacks, 10 m skipping, 15 air squats, 20 high knees and 10 m walking lunges) at 90% HR_max_ separated by 1 min walking, followed by a 1.5 min walking cool-down on the treadmill. The development of this HIIE protocol was based on prior research evaluating feasibility of real-world HIIE protocols designed for workplaces [28] and university environments [29] and current HIIE routines recommended by certified personal trainers in the community. The seated rest protocol consisted of watching a *National Geographic* video (Join this Man on a Safari to Sculpt Animals in the Wild; *National Geographic*). A research assistant measured HR during all three intervention conditions at time points that aligned with the transition periods from moderate intensity to high intensity (1 min, 3.5 min and 6 min), from high intensity to moderate intensity (2.5 min, 5 min and 7.5 min) and at the cessation of HIIE (9 min).

### 2.5. Statistical Analysis

Statistical analyses were conducted using SPSS (IBM, SPSS, v. 26 Chicago, IL, USA). With a sample size of 24 participants and a power of 0.88, the present study was sufficient to detect repeated measures effects computed using G * Power 3.1.9.6 [40]. Repeated measures ANOVA (significance set at *p* ≤ 0.05) were performed using Greenhouse–Geisser correction statistics with Bonferroni-corrected *t*-tests for post hoc comparisons. Reporting of main effects and interactions included partial η^2^ (0.01 small; 0.06 medium; 0.14 large effect size). Flanker RT and accuracy analyses were conducted utilizing a 3 (Condition: HIIE-aerobic, HIIE-aerobic/resistance and seated rest) × 2 (Time: 30 min and 85 min) × 2 (Type: Congruent and Incongruent) repeated measures ANOVA. Flanker interference scores for RT and accuracy were analyzed using a 3 (Condition: HIIE-aerobic, HIIE-aerobic/resistance and seated rest) × 2 (Time: 30 min and 85 min) model. P3 analyses for amplitude and latency were conducted using a 3 (Condition: HIIE-aerobic, HIIE-aerobic/resistance and seated rest) × 2 (Time: 30 min and 85 min) × 2 (Type: Congruent and Incongruent) × 5 (Site: Cz, CPz, Pz, POz and Oz) repeated measures ANOVA.

## 3. Results

The preliminary analyses were performed on session order to determine if order effects influenced outcomes associated with the treatment. The results did not reveal any significant interactions with mode (*F’s* (1,23) ≤ 1.79, *p’s* ≥ 0.15), across Flanker and P3 measures. Therefore, subsequent analyses were collapsed across session order. All mean (±SD) values for Flanker performance and P3 measures are reported in Table 1.

**Figure 1:**
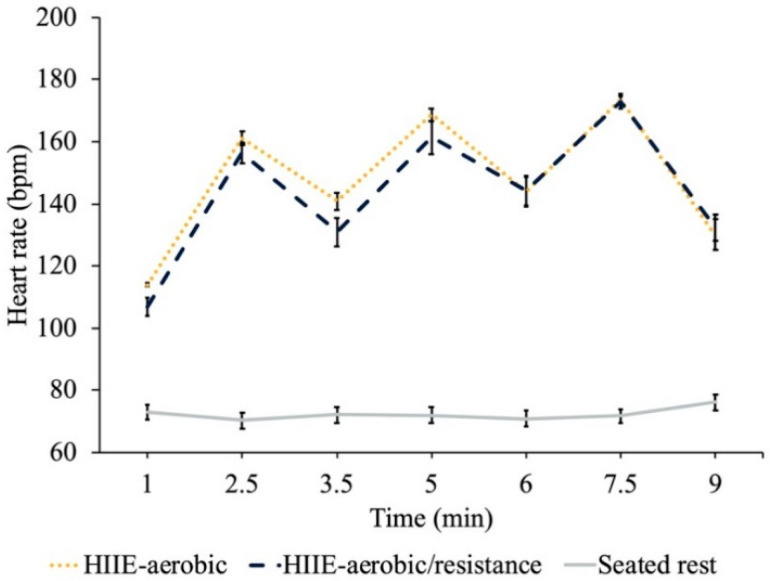
HR (bpm) results for each treatment condition measured at time intervals corresponding to the transition from moderate intensity to high intensity (1 min, 3.5 min and 6 min), from high intensity to moderate intensity (2.5 min, 5 min and 7.5 min) and at the cessation of HIIE (9 min).

The heart rate results revealed greater HR for the HIIE conditions compared to seated rest (*t’s* (23) ≥ 20.95, *p’s* ≤ 0.05), but no difference between HIIE conditions at each time point (*t’s* (23) ≤ 1.6, *p’s* ≥ 0.12) (see Figure 1).

### 3.1. Flanker Performance

#### 3.1.1. RT

The omnibus analysis for mean RT revealed a main effect of Type (*F* (1, 23) = 174.65, *p* < 0.01, η_p_^2^ = 0.88), which was superseded by a Time × Type interaction (*F* (1, 23) = 6.07, *p* < 0.01, η_p_^2^ = 0.21). Post hoc tests indicated shorter RT at 30 and 85 min for congruent trials compared to incongruent trials (*t’s* (23) ≥ 11.07, *p’s* ≤ 0.05). No significant difference was observed across time for congruent and incongruent trials (*t’s* (23) ≤ 1.34, *p’s* ≥ 0.19). Additionally, mean RT interference revealed a main effect of Time (*F* (1, 23) = 6.07, *p* = 0.02, η_p_^2^ = 0.21), revealing greater interference at 30 min compared to 85 min.

#### 3.1.2. Accuracy

The omnibus analysis for Flanker accuracy revealed a main effect of Time (*F* (1, 23) = 9.20, *p* < 0.01, η_p_^2^ = 0.29), indicating greater accuracy at 30 min compared to 85 min. Also, a main effect of Type (*F* (1, 23) = 37.71, *p* < 0.01, η_p_^2^ = 0.62) revealed greater accuracy for congruent trials compared to incongruent trials. Lastly, accuracy interference revealed no main effects or interactions (*F’s* (1, 23) ≤ 1.26, *p’s* ≥ 0.25).

### 3.2. ERP P3

#### 3.2.1. Latency

The omnibus analysis for P3 latency indicated a main effect of Type (*F* (1, 23) = 68.62, *p* < 0.01, η_p_^2^ = 0.75) and Site (*F* (1, 45.6) = 25.82, *p* < 0.01, η_p_^2^ = 0.53), which were superseded by a Type × Site interaction (*F* (2.0, 45.0) = 6.83, *p* < 0.01, η_p_^2^ = 0.23). The post hoc tests indicated shorter P3 latency for congruent compared to incongruent trials across all sites (*t’s* (23) ≥ 5.71, *p’s* ≤ 0.05). Lastly, the P3 latency revealed no main effects or interactions for Condition (*F’s* (1, 23) ≤ 2.02, *p’s* ≥ 0.09, η_p_^2^’s ≤ 0.08).

#### 3.2.2. Amplitude

**Figure 2:**
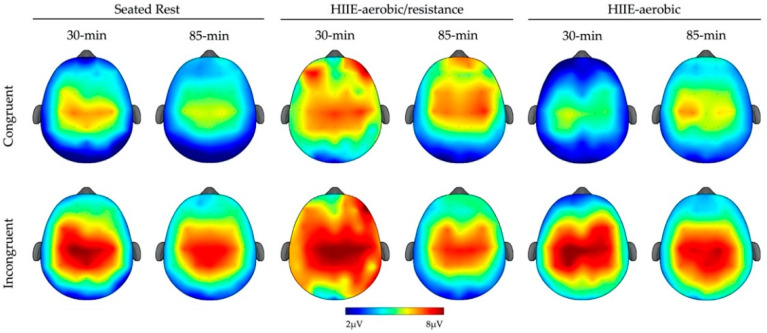
Topographic plots for P3 mean amplitude (300–600 ms) for each condition (columns), time (columns) and type (rows).

The omnibus analysis for P3 mean amplitude revealed a main effect of Type (*F* (1, 23) = 74.41, *p* < 0.01, η_p_^2^ = 0.76) and Site (*F* (1.6, 37.1) = 31.84, *p* < 0.01, η_p_^2^ = 0.58), which were superseded by a Type × Site interaction (*F* (2.5, 57) = 9.92, *p* < 0.01, η_p_^2^ = 0.30). The post hoc tests indicated larger P3 mean amplitude for incongruent compared to congruent trials across all sites (*t’s* (23) ≥ 5.21, *p’s* ≤ 0.05). Additionally, a Condition × Type interaction was observed (*F* (1.9, 43.9) = 6.32, *p* < 0.01, η_p_^2^ = 0.22). The decomposition of this interaction revealed larger P3 mean amplitude for incongruent trials compared to congruent trials across all conditions (*t’s* (23) ≥ 5.70, *p’s* ≤ 0.05). Lastly, a Time × Type interaction (*F* (1, 23) = 5.20, *p* < 0.01, η_p_^2^ = 0.18) revealed a larger P3 amplitude for incongruent trials compared to congruent trials across both time periods (*t’s* (23) ≥ 6.47, *p’s* ≤ 0.05) (see Figure 2).

## 4. Discussion

The present study evaluated inhibitory control and P3 following different HIIE conditions across an extended period. In contrast to our a priori hypotheses, these data suggest that neither HIIE-aerobic nor HIIE-aerobic/resistance enhance or inhibit behavioral and neural markers of cognitive functioning beyond 30 min compared to seated rest.

The present behavioral results are inconsistent with recent meta-analytical findings [23,31], which suggest a small but significant improvement in inhibitory control following continuous and intermittent high-intensity exercise compared to rest. These inconsistencies could be due to the time of cognitive assessment and duration of the HIIE protocol. Longer exercise bouts may be necessary to observe significant delayed effects, as evident in research by Tsukamoto et al. [10], which revealed cognitive improvements immediately after and 30 min after a 33 min bout of HIIE. Furthermore, previous HIIE studies have only assessed cognitive function immediately after and at 20 and 30 min intervals following HIIE [8,9,10]. Hence, it is possible that the duration of the HIIE bout influences the duration of the cognitive benefits, and that these benefits only last for a short period. Although our data do not reflect these findings, they do complement existing literature, which suggests that short HIIE bouts do not interfere with inhibitory control performance after a delay of 30 min or longer. Taken together, future HIIE studies should consider the short window of potential cognitive benefits in light of the present findings.

Another possible explanation for this lack of replication of previous behavioral results concerns individual differences in psychological factors related to mental context experienced prior to and during the exercise bout (e.g., incompetence, affect, motivation and self-efficacy). HIIE exercise is a difficult routine to accomplish, especially for those unfamiliar or inexperienced, and this may increase heterogeneity in psychological preferences and attitudes toward the task [41]. This assertion is supported by emerging evidence suggesting that individual differences in cognitive and affective mood states moderate psychological performance following the exercise bout [24,42,43,44,45]. Although the present study did not include measures of the psychological mental context surrounding the exercise bouts, it is likely that such confounding factors may have contributed to the null findings observed in the present study. Future work may benefit from evaluating preference-based exercise routines—as opposed to prescription-based, experimenter-controlled routines—to reduce the heterogeneity of psychological factors that may influence cognitive behavior changes in response to acute exercise. 

Regarding P3, the present findings revealed maintenance of P3 latency and amplitude regardless of the HIIE condition. These data are not in line with our hypotheses and are contrary to the findings of similar protocols revealing decreases in P3 amplitude after acute HIIE [8,9]. For instance, Kao et al. [9] found an increase in P3 amplitude following moderate-intensity continuous physical activity but not following HIIE, and observed a decrease in P3 latency after HIIE. The authors suggest that moderate-intensity physical activity may increase attentional-resource allocation to facilitate cognitive demands. The authors further suggest that the effects of HIIE may facilitate a down-regulation in attentional resources required to facilitate faster information processing needed to perform a demanding, goal-directed cognitive task efficiently. However, despite similar protocols, our data did not reveal such a modulation in brain function at 30 and 85 min following HIIE, contrary to our predictions that the P3 measures would follow a similar trend. As with the behavior results, it is possible that any positive benefits to P3 following HIIE are limited in duration and return to baseline less than 30 min after HIIE.

Despite the novelty of the present investigation, limitations are present. First, as addressed previously, the duration of the HIIE bouts may have contributed to the null findings. Future research should include protocols with varied timing to determine if duration moderates the longevity of cognitive benefits post-exercise. Secondly, cognitive and neuroelectrical measurements were not performed immediately after the HIIE and rest conditions. Our protocol may have elicited cognitive improvements that returned to baseline prior to the first assessment. Nevertheless, these data do not support a persistent timing effect for improved cognition and brain functioning associated with HIIE. Therefore, further research is needed to determine cognitive task timing effects following different exercise modalities. Next, the different HIIE modalities were primarily aerobic in nature with the HIIE-aerobic/resistance protocol incorporating arguably only two resistance activities (i.e., walking lunges and air squats). Given that the HR data revealed no difference in intensity, it is possible that both conditions constituted high-intensity aerobic exercise. Lastly, no pre-test assessment was conducted to control for day effects across each testing condition. However, participants in the present study were counterbalanced across sessions with the seated rest condition serving as the control. Regardless, future research should include a baseline measure to mitigate timing limitations, both for duration of the physical activity protocol and the timing of cognitive task performance.

## 5. Conclusions

In conclusion, these data suggest that inhibitory control and neuroelectric underpinnings are not affected by different modalities of HIIE after 30 min. However, our findings do have useful implications for considering time-efficiency in relation to exercise, and for promoting short bouts of physical activity that do not interfere with cognitive functioning. The findings from this investigation expand the current understanding regarding acute effects of HIIE on cognition, which is important for future research addressing public health concerns from a psychological and physiological perspective in relation to HIIE. HIIE is a time-efficient physical activity modality that may be tailored to individual fitness levels, and which can improve cardiorespiratory fitness and reduce the risks of cardiovascular disease and Type II diabetes. Therefore, the current findings warrant additional investigation to further examine the impacts on cognition and other public health risks following acute bouts of HIIE to improve optimal health and well-being.

## Figures and Tables

**Table 1:** Mean (±SD) values for Flanker performance and P3.

	Seated Rest		HIIE-Aerobic/Resistance		HIIE-Aerobic	
Measure	30 min	85 min	30 min	85 min	30 min	85 min
Flanker Accuracy (%)						
Congruent	98.3 ± 2.3	96.6 ± 3.5	98.1 ± 2.8	97.2 ± 2.8	98.3 ± 1.9	96.0 ± 5.6
Incongruent	90.0 ± 9.9	88.8 ± 7.6	91.3 ± 5.9	90.5 ± 6.2	91.1 ± 7.2	88.6 ± 8.2
Interference	8.4 ± 9.4	7.4 ± 6.6	6.8 ± 5.3	6.5 ± 6.4	6.8 ± 6.8	7.0 ± 5.5
Flanker RT (ms)						
Congruent	401.3 ± 39.6	390.9 ± 32.9	392.3 ± 40.3	397.2 ± 41.0	398.8 ± 35.4	401.5 ± 39.3
Incongruent	447.3 ± 42.9	431.9 ± 39.8	439.6 ± 45.9	440.0 ± 42.7	445.9 ± 44.2	442.8 ±46.3
Interference	46.0 ± 17.7	41.0 ± 20.0	47.4 ± 18.8	42.8 ± 19.6	47.1 ± 17.3	41.3 ± 18.9
P3 congruent amplitude (µV)						
Cz	6.4 ± 4.0	5.1 ± 2.9	5.4 ± 3.1	5.4 ± 2.4	4.9 ± 3.3	5.3 ± 3.4
CPz	6.3 ±3.8	5.1 ± 2.8	4.7 ± 3.2	4.8 ± 2.2	4.8 ± 3.2	5.2 ± 3.2
Pz	4.5 ± 3.9	3.3 ± 2.6	3.2 ± 3.6	3.0 ± 2.7	3.4 ± 3.2	4.0 ± 3.0
POz	3.3 ± 3.7	3.7 ± 7.6	2.1 ± 3.5	2.5 ± 2.9	2.5 ± 3.6	2.9 ± 4.0
Oz	2.5 ± 2.5	1.9 ± 2.4	1.2 ± 3.2	2.9 ± 4.2	1.9 ± 2.5	2.8 ± 2.9
P3 congruent latency (ms)						
Cz	368.8 ± 61.4	357.3 ± 37.0	365.3 ± 57.4	366.0 ± 61.7	361.2 ± 63.6	361.5 ± 44.2
CPz	371.4 ± 61.2	354.7 ± 37.6	359.3 ± 56.9	355.7 ± 52.3	355.7 ± 39.2	359.8 ± 43.3
Pz	365.1 ± 57.8	344.0 ± 36.5	358.3 ± 51.3	345.8 ± 34.8	341.0 ± 37.6	351.6 ± 43.5
POz	365.2 ± 68.5	347.6 ± 36.7	352.3 ± 47.0	343.7 ± 34.0	332.8 ± 21.7	350.0 ± 44.0
Oz	359.0 ± 59.6	344.8 ± 33.6	344.0 ± 41.4	350.9 ± 47.9	328.6 ± 17.6	344.4 ± 41.1
P3 incongruent amplitude (µV)						
Cz	7.7 ± 4.3	6.7 ± 3.1	7.5 ± 3.4	6.7 ± 3.3	7.7 ± 3.5	7.1 ± 3.3
CPz	7.7 ± 4.2	6.9 ± 3.2	6.8 ± 3.4	6.2 ± 2.9	7.6 ± 3.5	7.0 ± 3.2
Pz	5.9 ± 4.2	5.1 ± 3.1	5.0 ± 3.7	4.3 ± 3.2	6.1 ± 3.5	6.1 ± 3.4
POz	4.5 ± 4.0	4.6 ± 4.8	4.0 ± 3.3	3.6 ± 3.1	4.8 ± 3.6	4.8 ± 3.9
Oz	3.3 ± 2.7	2.7 ± 2.2	2.7 ± 3.2	3.5 ± 4.0	3.6 ± 2.4	4.1 ± 3.0
P3 incongruent latency (ms)						
Cz	407.8 ± 50.2	406.1 ± 45.0	410.5 ± 48.5	413.9 ± 58.6	409.5 ± 43.4	404.7 ± 40.8
CPz	405.6 ± 51.9	401.5 ± 44.9	403.3 ± 46.9	403.9 ± 59.5	408.2 ± 43.3	400.3 ± 47.3
Pz	392.4 ± 57.3	379.4 ± 44.7	404.3 ± 53.3	383.9 ± 53.6	386.8 ± 40.5	392.0 ± 45.2
POz	387.2 ± 58.6	368.3 ± 48.5	383.6 ± 56.0	368.1 ± 50.8	379.0 ± 40.7	379.1 ± 52.7
Oz	384.9 ± 57.8	367.3 ± 37.0	368.0 ± 43.6	369.7 ± 43.3	371.8 ± 45.5	374.0 ± 49.8

Note: ms = milliseconds; RT = reaction time; Accuracy interference = congruent accuracy—incongruent accuracy; RT interference = incongruent reaction time—congruent reaction time.
